# Fungi-Bacteria Associations in Wilt Diseased Rhizosphere and Endosphere by Interdomain Ecological Network Analysis

**DOI:** 10.3389/fmicb.2021.722626

**Published:** 2021-09-06

**Authors:** Lin Tan, Wei-ai Zeng, Yansong Xiao, Pengfei Li, Songsong Gu, Shaolong Wu, Zhengguang Zhai, Kai Feng, Ye Deng, Qiulong Hu

**Affiliations:** ^1^Hunan Agricultural University, Changsha, China; ^2^Changsha Tobacco Company of Hunan Province, Changsha, China; ^3^Chenzhou Tobacco Company of Hunan Province, Chenzhou, China; ^4^Wenshan Tobacco Company of Yunnan Province, Wenshan, China; ^5^CAS Key Laboratory for Environmental Biotechnology, Research Center for Eco-Environmental Sciences, Chinese Academy of Sciences, Beijing, China; ^6^Institute for Marine Science and Technology, Shandong University, Qingdao, China; ^7^Tobacco Company of Hunan Province, Changsha, China; ^8^College of Resources and Environment, University of Chinese Academy of Sciences, Beijing, China

**Keywords:** bacterial wilt invasion, soil microbiota, endophytic microbiota, molecular ecological network, biocontrol fungal resources

## Abstract

In the plant rhizosphere and endosphere, some fungal and bacterial species regularly co-exist, however, our knowledge about their co-existence patterns is quite limited, especially during invasion by bacterial wilt pathogens. In this study, the fungal communities from soil to endophytic compartments were surveyed during an outbreak of tobacco wilt disease caused by *Ralstonia solanacearum*. It was found that the stem endophytic fungal community was significantly altered by pathogen invasion in terms of community diversity, structure, and composition. The associations among fungal species in the rhizosphere and endosphere infected by *R. solanacearum* showed more complex network structures than those of healthy plants. By integrating the bacterial dataset, associations between fungi and bacteria were inferred by Inter-Domain Ecological Network (IDEN) approach. It also revealed that infected samples, including both the rhizosphere and endosphere, had more complex interdomain networks than the corresponding healthy samples. Additionally, the bacterial wilt pathogenic *Ralstonia* members were identified as the keystone genus within the IDENs of both root and stem endophytic compartments. *Ralstonia* members was negatively correlated with the fungal genera *Phoma, Gibberella*, and *Alternaria* in infected roots, as well as *Phoma, Gibberella*, and *Diaporthe* in infected stems. This suggested that those endophytic fungi may play an important role in resisting the invasion of *R. solanacearum.*

## Introduction

*Ralstonia solanacearum*, the causative agent of soil-borne bacterial wilt disease in plants, is often found in agricultural land used for tobacco cultivation. Once the pathogen invades the plant root system, it rapidly spreads to the stem, causing an internal system imbalance of the entire tobacco plant, accelerating senescence and death (Li et al., 2017; Wei et al., 2018). As important members of the plant microecosystem (Nilsson et al., 2019), fungi play a vital role in promoting the material cycle of agro-ecosystem and affecting plant growth and health (Lilleskov et al., 2011; Zhang et al., 2021). Therefore, dynamic changes in the structure and composition of the plant soil fungal community can indicate the alterations of soil micro-ecological environment (Shi et al., 2020). Plant endophytic fungi exist within the host plant and interact closely with other microorganisms to promote plant growth, resist the invasion of plant pathogens, and improve the disease resistance of host plant through their own metabolites or induction of the host’s metabolites (Arnold, 2007; Mousa et al., 2016; Bastias et al., 2017). However, our knowledge about how the soil and endophytic fungal communities change under the invasion of *R. solanacearum* is quite limited.

With the surge of research on microbial communities in various ecological environments, the interdomain relationships between different types of microbial communities has attracted great attention (Kapitan et al., 2019; Wang et al., 2020). The association of plant bacterial and fungal communities is critical to overall microbial community structure and plant health (Hassani et al., 2018), and intrigues numerous botanists and microbiologists. Plant microbial communities live and colonize several zones including the bulk soil, rhizosphere soil, phyllosphere, and endosphere (Bulgarelli et al., 2013; Kumar et al., 2017). They play a vital role in the acquisition of nutrients by plants, mutual defense, and co-evolution (Martin et al., 2017; Fitzpatrick et al., 2018). Understanding the relationship between related microorganisms in natural ecosystems may help us better explore and learn about the assembly, diversity, and stability of plant-related communities (Haq et al., 2014; van der Putten, 2017). Recently, researchers have attempted to identify plant-related microbial communities from bacteria to fungi, as well as their associations, by means of microbiome annotation database or molecular-based experimental methods, broadening our basic knowledge of these types of microbial communities (Jackrel et al., 2017; Bamisile et al., 2018; Levy et al., 2018). Endophytic fungi are often considered to be beneficial to their host plants (Qian et al., 2019), because they may play various ecological roles such as promoting plant growth, enhancing the absorption of nutrient, resistance against various plant pathogens, as well as tolerance against various biotic and abiotic stresses (Waqas et al., 2012; Jia et al., 2016; Terhonen et al., 2016; Ripa et al., 2019). Current studies on tobacco wilt, a bacterial disease, mainly focus on the diversity and structural composition of the tobacco soil microbial community and its correlation with soil physicochemical properties, as well as the associations between the pathogen and bacterial species (Jiang et al., 2017; Li et al., 2017). However, the associations between the closely related bacterial and fungal communities in various zones of the plant-soil microecosystem under the invasion of *R. solanacearum* and the roles of endophytic fungi remains unclear.

The purposes of the current study are to: (i) Illuminate the characteristics of fungal communities in the bulk and rhizosphere soils and the root and stem endophytic compartments of healthy tobacco plants and those infected by *R. solanacearum*; (ii) Reveal the associations among species of fungal communities from various zones of the plant-soil microecosystem via molecular ecological network analysis; (iii) Explore the associations between fungi and bacteria through interdomain ecological network (IDEN) analysis; (iv) Study the associations between pathogenic *Ralstonia* members and fungi through sub-network analysis, and to explore fungal biocontrol resources that may antagonize the bacterial wilt pathogen. From the obtained results, we will provide a new strategy and theoretical support for enriching the study of tobacco endophytic fungal resources and for exploring the antagonistic fungal resources targeting *R. solanacearum.*

## Materials and Methods

### Sample Collection and Processing

The general collecting locations and methods of collecting and processing for soil and endophytic samples have been described previously in detail (Hu Q.L. et al., 2020), and will only be summarized here. Detailed location and other information were list in Supplementary Table 1. Eighty samples of bulk soil, rhizosphere soil, roots, and stems for tobacco cultivar Yunyan 87, including healthy and severely infected by *R. solanacearum* (grade 5–9 infection) (Chinese Standards, 2008), were collected from 5 different tobacco field sites located in the Chenzhou tobacco-growing region of Hunan province of China on June 2016 (mature stage of tobacco). The samples included 10 bulk soils samples of healthy tobacco (HBS), 10 bulk soil samples of wilt-infected tobacco (IBS), 10 rhizosphere samples of healthy tobacco (HRS), 10 rhizosphere samples of wilt-infected tobacco (IRS), 10 root samples of healthy tobacco (HR), 10 root samples of wilt-infected tobacco (IR), 10 stem samples of healthy tobacco (HS), 10 stem samples of wilt-infected tobacco (IS). Bulk soil samples were collected by shaking soil off tobacco roots. After shaking off bulk soils, the rhizosphere soils adhering to plant roots were collected in 50 mL tubes containing PBS (0.1% Tween 80) with a brush. After stirring for 5 min, the resulting suspension was then poured into a sterile centrifuge tube, this process was repeated a further two times. The suspensions were mixed and centrifuged for 5 min at 2,000 × *g*. The resulting sediment pellets were collected as the rhizosphere soils, which were stored at –80°C until DNA extraction. The roots and stems were processed immediately by washing consecutively with 75% ethanol, 2.5% sodium hypochlorite, and sterile water. They were then cut into small pieces and homogenized using a mortar and pestle with PBS, then transferred into a centrifuge tube for further treatment. Ultimately, the resulting sediment pellets from soil samples and resulting cell pellet from endophytic samples were stored in a freezer at –80°C until DNA extraction.

### DNA Extraction and High-Throughput Sequencing

Total DNA was extracted in duplicate using the FastDNATM SPIN kit (MP Biomedicals) following the manufacturer’s instructions. Methods of PCR amplification and high-throughput sequencing for 16S rRNA genes followed those of Zhang et al. (2017). The V5-V6 region of 16S rRNA gene was amplied by the 799F (5′-AACMGGATTAGATACC CKG-3′)/1115R (5′-AGGGTTGCGCTCGTTG-3′) primers, and ITS2 fragment was amplified using the primer pair 5.8F (5′-AACTTTYRRCAAYGGAT CWCT-3′)/4R (5′-AGCCTCCGCTTATTGATATGCTTAART-3′) (Taylor et al., 2016). PCR amplification was performed in a 50 μL reaction system including 5 μL DNA template, 2.5 U of Taq DNA Polymerase (TaKaRa), 1 × Taq buffer, 75 μM dNTP and 0.3 μM of each primer. The thermal cycle operations for ITS2 fragment was performed as follows: 94°C for 1 min; following 35 cycles of 94°C for 20 s, 57°C for 25 s, and 68°C for 45 s, with a final elongation step at 68°C for 10 min, and finally stored at 4°C. The recovered products were qualified and quantified by a NanoDrop Spectrophotometer (Nano-100, Aosheng Instrument Co., Ltd.). Subsequently, the purified amplicons were pooled together and sequenced on Miseq sequencing machine (Illumina) at Central South University, Changsha, China.

### Sequence Processing and Analysis

Preprocessing of raw reads of 16S rRNA genes and ITS2 fragment were submitted to an in-house pipeline^1^ integrated with various bioinformatics tools (Feng et al., 2017). All reads were assigned to individual sample according to their barcodes, allowing for a single mismatch. After trimming off the barcode and primer sequences, the pair-ended sequences for 16S rRNA genes were merged and their quality was checked by Flash program (Kong, 2011). The pair-ended sequences for ITS2 fragment with forward and reverse primers combinations were trimmed off. Subsequently, the sequences were passed through the ITSx program to remove the ITS flanking regions and non-fungal sequences (Bengtsson-Palme et al., 2013). Next, sequences were clustered into operational taxonomic units (OTUs) using UPARSE (SRP101823 for 16S rRNA gene sequences, and SRP123067 for ITS sequences) with a 97% sequence similarity threshold (Edgar, 2013). Ultimately, an OTU table was created and the total read counts were resampled before use in downstream analyses.

### Statistical Analysis

The statistical significance of differences between two groups were tested by Wilcoxon test. Two measurements of alpha-diversity, Richness and Chao1, were calculated to assess the diversity of fungal communities. Richness was obtained by counting the number of species displayed in the OTU table. The Chao1 value was calculated using Mothur software (Chao, 1984; Schloss et al., 2009). Principal coordinate analysis (PCoA) was used to analyze the β-diversity of fungal communities in the bulk soil, rhizosphere soil, and root and stem endophytic compartments for both healthy and infected plants. Dissimilarity tests for soil and endophytic microbial community structures between healthy and infected samples were performed by using PERMANOVA based on Jaccard distance. Differences in soil and plant endophytic community compositions from healthy and infected samples were determined using an analysis of variance (ANOVA).

### Random Matrix Theory Based Molecular Ecology Networks

To reveal the associations among fungal species in soil and endophytic fungal communities, from both healthy and infected samples, we constructed phylogenetic molecular ecological networks (MEN) via a Random Matrix Theory (RMT)-based approach (Deng et al., 2012) in molecular ecological network analysis pipeline (MENA)^2^ (Deng et al., 2016).

### Interdomain Ecological Network Construction

The topology of ecological networks can represent the assembly process of microbial communities (Layeghifard et al., 2017), and the connections between interacting species can be used to predict ecosystem stability (Thebault and Fontaine, 2010). Recently, Feng et al. (2019) set up a workflow to construct IDEN, to find the association between two taxonomic groups (i.e., aboveground plants and underground bacteria) in ecological surveys. This method provided technical support for our analysis of the interdomain microbial associations between fungal and bacterial communities of soil and endophytes.

To elucidate associations between fungi and bacteria in soil and endophytic communities, by integrating the bacterial dataset (NCBI SRA database, accession PRJNA540089), interdomain ecological networks via SparCC approaches based on the inter-domain ecological network analysis pipeline^3^ workflow (Feng et al., 2019) were constructed. The threshold value for generating regional IDEN was 0.30, with 0.05 significance, to filter the non-correlated associations. The obtained adjacent matrix associated with the bipartite graph consisted of 1 or 0, showing presence/absence of corresponding fungi-bacteria association. The topological properties (connectance, links per species, specialization asymmetry, and web asymmetry) were calculated to explore alterations in associations between fungi and bacteria in the soil and endophytic communities under the invasion of *R. solanacearum*. The SparCC method with default parameters (Feng et al., 2019) was used for correlation analysis of specific associations between the pathogen and fugal members. The constructed networks were visualized using Gephi 0.9.2 software (Bastian et al., 2009). The keystone microorganisms were identified by the Zi-Pi plot based on the nodes’ roles within their own network (Deng et al., 2012).

## Results

### Effects of Wilt Pathogen Invasion on the Fungal Community Diversity and Structure of Soil and Tobacco Endophyte

**FIGURE 1:**
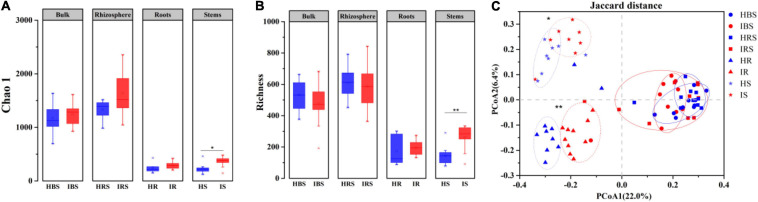
Diversity and structure of fungal communities in the healthy and infected bulk soil, rhizosphere soil, and root and stem endophytic compartments based on internal transcribed spacer (ITS) sequencing. **(A)** Richness. **(B)** Chao 1 index. **(C)** PCoA plot of fungal communities in healthy and infected plants. Note: blue and red indicate healthy and infected samples, respectively. HBS: bulk soils samples of healthy tobacco, IBS: bulk soil samples of wilt-infected tobacco, HRS: rhizosphere samples of healthy tobacco, IRS: rhizosphere samples of wilt-infected tobacco, HR: root samples of healthy tobacco, IR: root samples of wilt-infected tobacco, HS: stem samples of healthy tobacco, IS: stem samples of wilt-infected tobacco. Wilcoxon test and PERMANOVA were used to examine the statistical significance of differences for alpha and beta diversity, respectively. Difference was significant at *, 0.05; **, 0.01.

Using high-throughput sequencing, a total of 8, 317 operational taxonomic units (OTUs) were obtained from 80 samples, 7, 216 OTUs among the soil samples and 2, 786 OTUs among the endophytic samples. Both observed Chao1 (Figure 1A) and estimated richness (Figure 1B) demonstrated no significant difference in fungal community diversity between healthy and infected tobacco plants regarding the bulk soil, rhizosphere soil, and root endophytes. However, the fungal richness of infected stems was significantly higher than that of healthy counterparts (Wilcoxon test, *P* < 0.05). As shown in Figure 1C, the endophytic fungal communities for healthy and infected plants were clearly separated in the PCoA plot, while the bulk, and rhizosphere soil communities of healthy and infected plants partially overlapped, indicating significant differences in fungal community structure between the healthy and infected endophytic compartments (PERMANOVA, *P* < 0.05).

### Effects of Wilt Pathogen Invasion on the Fungal Community Composition of Soil and Tobacco Endophyte

**FIGURE 2:**
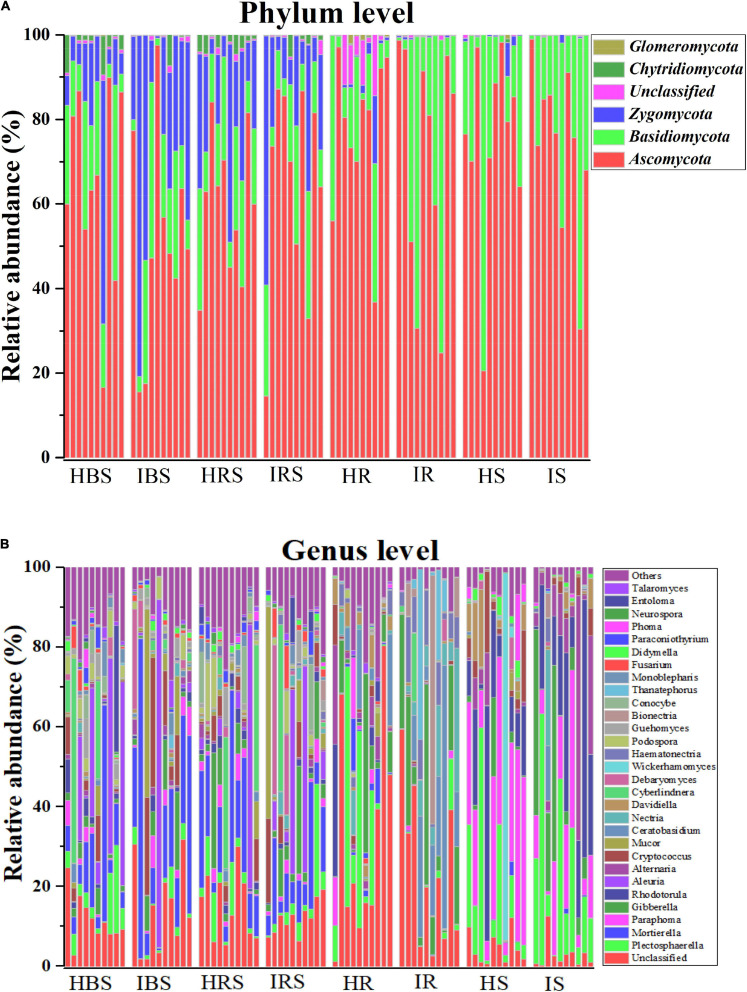
Comparison of the community compositions of soil and plant endophytic fungi from healthy and infected samples. **(A)** Comparison of the community compositions of the healthy and infected bulk soil, rhizosphere soil, and root and stem endophytic compartments with relative abundance > 1% OTUs at the phylum level. **(B)** Comparison of the community compositions of the healthy and infected bulk soil, rhizosphere soil, and root and stem endophytic compartments with relative abundance > 1% OTUs at the genus level.

The composition of tobacco soils and endophytic fungal communities at the phylum level is shown in Figure 2A (with relative abundance > 1%). In the soil communities, the most abundant fungal phyla were *Ascomycota*, *Basidiomycota*, and *Zygomycota*, followed by *Chytridiomycota* and *Glomeromycota*. In the tobacco endophytic fungal communities, the most abundant fungal phyla were *Ascomycota* and *Basidiomycota*, followed by *Zygomycota*, *Chytridiomycota*, and *Glomeromycota*. The composition of tobacco soil and endophytic fungal communities at the genus level is shown in Figure 2B (with relative abundance > 1%). In the soil communities, the fungal genera with higher relative abundance included *Mortierella, Aleuria, Cyberlindnera, Cryptococcus, Plectosphaerella, Gibberella, Mucor, Debaryomyces, Podospora, Entoloma, Paraphoma, Rhodotorula, Fusarium, Conocybe*, and *Guehomyces.* In the tobacco endophytic fungal communities, the fungal genera with higher relative abundance included *Plectosphaerella, Paraphoma, Gibberella, Rhodotorula, Alternaria, Ceratobasidium, Nectria, Davidiella, Haematonectria, Bionectria*, and *Thanatephorus.* The genera whose abundance increased significantly in the infected soil samples as compared to healthy soil samples were *Gibberella, Cryptococcus, Mucor, Nectria, Debaryomyces*, and *Haematonectria*, and the genera that displayed significantly deceased abundance were *Rhodotorula, Ceratobasidium, Cyberlindnera, Podospora, Conocybe, Monoblepharis, Paraconiothyrium*, and *Phoma*. The genera whose abundance increased significantly in the endophytic communities of infected samples compared to those of healthy samples were *Haematonectria, Gibberella, Ceratobasidium, Nectria, Bionectria*, and *Didymella*, and the genera with significantly deceased abundance were *Cryptococcus, Didymella, Mortierella, Paraphoma, Davidiealla, Phoma*, and *Mucor*. In summary, the fungal community compositions and relative abundance were different between the healthy and infected soil communities, between the healthy and infected endophytic communities, and between the soil and endophytic communities (Supplementary Table 2, *P* < 0.05).

### The Species Associations Among Fungal Communities

**FIGURE 3:**
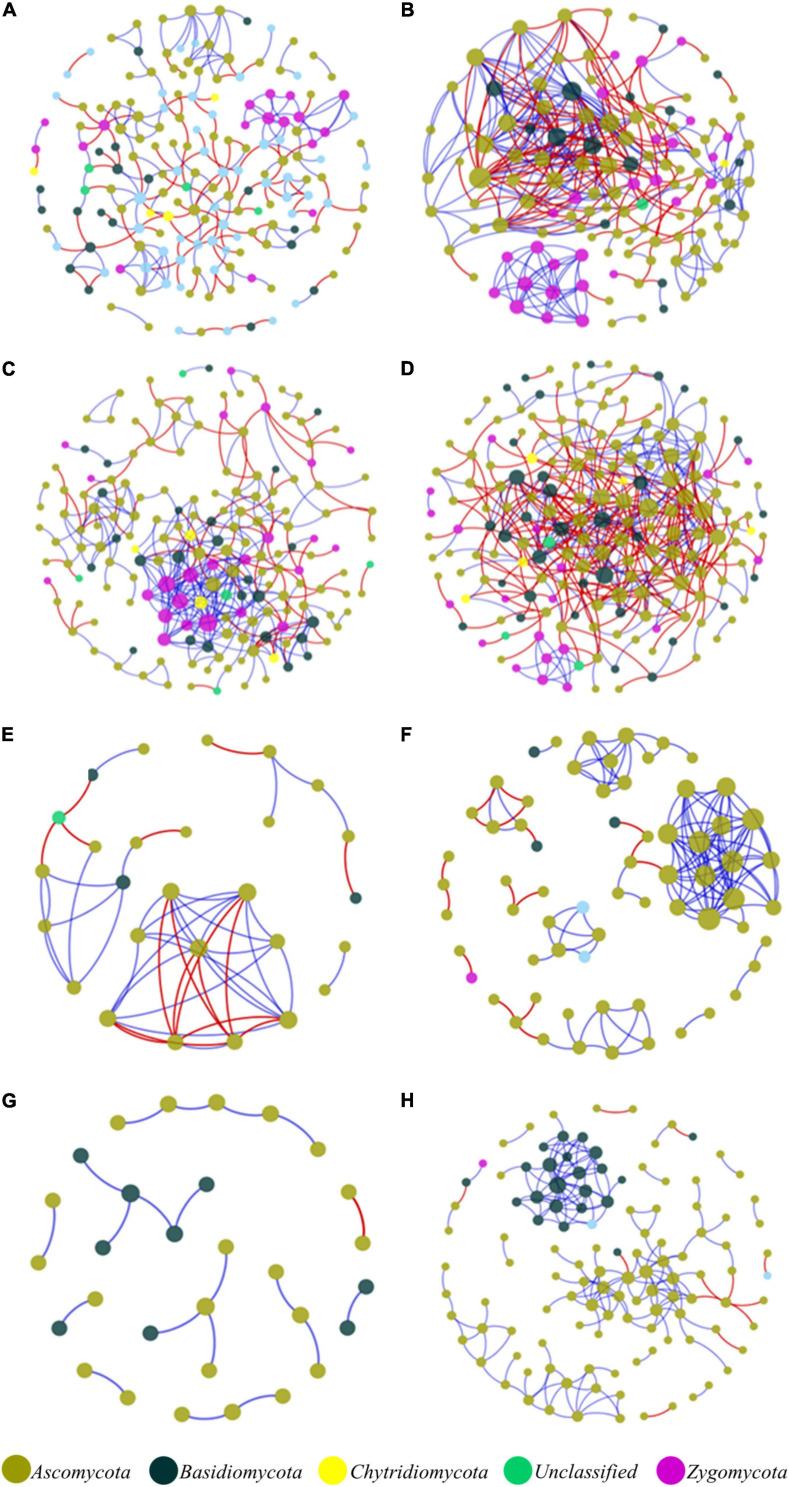
Molecular ecological networks of microorganism associations in the fungal communities of healthy and infected samples. **(A)** Healthy bulk soil. **(B)** Infected bulk soil. **(C)** Healthy rhizosphere soil. **(D)** Infected rhizosphere soil. **(E)** Healthy root endophytic compartment. **(F)** Infected root endophytic compartment. **(G)** Healthy stem endophytic compartment. **(H)** Infected stem endophytic compartment. Node color represents phylum-level taxonomy. Blue links indicate positive correlations between nodes, and red links indicate negative correlations.

In the network structures of rhizosphere soil, root endophytes, and stem endophytes, the fungal network structures of the infected samples showed a higher complexity and more links than those of healthy samples (Table 1). For example, the number of network nodes and links of the infected rhizosphere soil were 187 and 518, respectively, and those of the healthy rhizosphere soil were 179 and 367, respectively. In addition, the average clustering coefficients (avgCCs) of the empirical networks of all tested samples (0.02–0.67) were higher than those of the corresponding random networks (0.01–0.228), suggesting that the eight constructed networks all had typical small-world network characteristics (Watts and Strogatz, 1998). The modularity (*M*) values of all empirical networks (0.534–0.91) were significantly higher than the *M*-values of the corresponding random networks (0.339–0.860), indicating modular topological features of constructed networks (Newman, 2016). The visualized networks were constructed to intuitively display the associations among microorganisms in the fungal communities of healthy and infected samples, as shown in Figure 3. In general, there were obvious differences in the network topological structure between infected and healthy samples from all four of the investigated microecosystem zones.

**TABLE 1:** Topological features of the soil and endophytic fungal community networks in healthy and infected samples.

Empirical networks									Random networks		
Samples	Similarity threshold	Total nodes	Total links	*R* ^2^	Average degree (avgK)	Average path distance (GD)	Average clustering coefficient (avgCC)	Modularity: (module⋅no)	Average clustering coefficient (avgCC)	Modularity: (module⋅no)	Average path distance (GD)
HBS	0.85	174	232	0.76	2.67	7.42	0.28	0.81 (21)	0.034 ± 0.01	0.67 ± 0.01	5.22 ± 0.16
IBS	0.85	133	368	0.74	5.53	4.14	0.42	0.91 (24)	0.01 ± 0.02	0.82 ± 0.01	6.57 ± 1.06
HRS	0.85	179	367	0.87	4.10	6.33	0.32	0.67 (15)	0.06 ± 0.02	0.52 ± 0.01	4.08 ± 0.12
IRS	0.85	187	518	0.80	5.54	4.26	0.29	0.54 (16)	0.11 ± 0.02	0.44 ± 0.01	3.58 ± 0.08
HR	0.90	27	49	0.79	3.63	1.90	0.50	0.534 (4)	0.228 ± 0.058	0.339 ± 0.018	2.56 ± 0.11
IR	0.90	66	138	0.69	4.18	2.07	0.67	0.653 (12)	0.188 ± 0.0278	0.383 ± 0.014	3.04 ± 0.12
HS	0.90	30	20	0.99	1.33	1.62	0.02	0.865 (10)	0.01 ± 0.002	0.860 ± 0.016	1.67 ± 0.18
IS	0.90	119	200	0.76	3.36	3.48	0.45	0.750 (19)	0.08 ± 0.02	0.522 ± 0.015	3.70 ± 0.11

*HBS, bulk soils samples of healthy tobacco; IBS, bulk soil samples of wilt-infected tobacco; HRS, rhizosphere samples of healthy tobacco; IRS, rhizosphere samples of wilt-infected tobacco; HR, root samples of healthy tobacco; IR, root samples of wilt-infected tobacco; HS, stem samples of healthy tobacco; IS, stem samples of wilt-infected tobacco.*

### IDEN Between Fungal and Bacterial Communities

**FIGURE 4:**
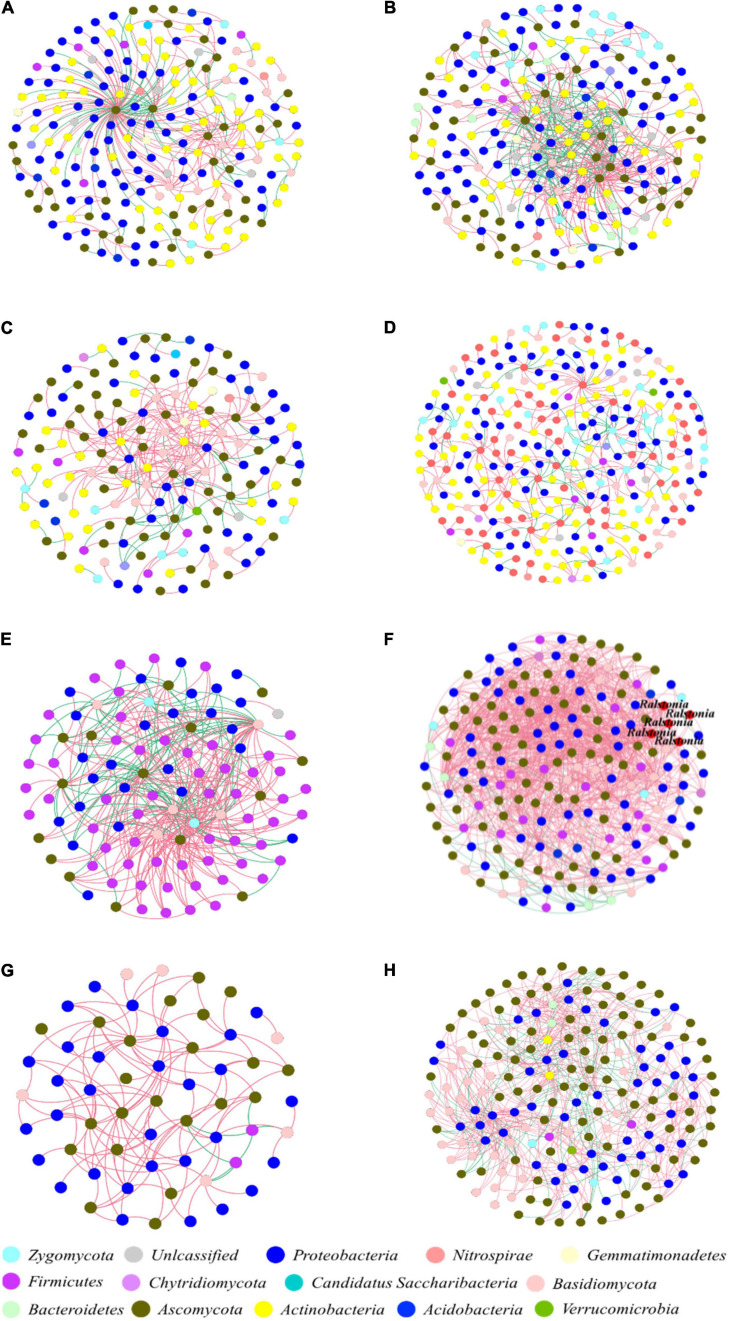
Interdomain ecological networks of the bacterial-fungal associations of healthy and infected samples. **(A)** Healthy bulk soil. **(B)** Infected bulk soil. **(C)** Healthy rhizosphere soil. **(D)** Infected rhizosphere soil. **(E)** Healthy root endophytic compartment. **(F)** Infected root endophytic compartment. **(G)** Healthy stem endophytic compartment. **(H)** Infected stem endophytic compartment. Node color indicates phylum-level taxonomy, and the pathogen *Ralstonia* was labeled at the genus level. Blue links indicate positive correlations between nodes, and red links indicate negative correlations.

The eight constructed networks of fungi-bacteria associations in healthy and infected samples from the surrounding bulk soil, rhizosphere soil, and root and stem endophytic compartments showed some basic bipartite topological structures, e.g., the modular structure and high web asymmetry of two species groups (Table 2), and all showed significant topological differences (Figure 4). According to the topological indices of the networks (Table 2), the total numbers of network nodes and links of the infected samples were all higher than the corresponding healthy samples, indicating more complex and compact bacterial-fungal associations in the infected samples. The higher value of web asymmetry for networks of infected root and stems endophytes showed that more fungi were involved in the interdomain associations. Instead of higher positive and negative associations for soil samples, the IDENs contained large proportions of negative associations in infected root and stem endophytic compartments compared to healthy samples, with more fungal nodes involved (1,298 vs. 266 for roots, and 493 vs. 131 for stems), suggesting resistance relationships between bacteria and fungi. Module detection analysis further demonstrated smaller modularity and fewer modules for IDENs of the endophytic compartments, implying closer fungi-bacteria associations. In addition, we found the greatest number of network links and the highest number of links per species in the IDEN of infected root endophytic compartment, indicating larger proportions of bacterial-fungal associations among the infected root endophytes.

**TABLE 2:** Interdomain network topology features of healthy and infected soil and endophytic microbial communities.

	HBS	IBS	HRS	IRS	HR	IR	HS	IS
No. of bacteria	157	142	86	185	82	91	32	59
No. of fungi	55	72	78	114	23	96	28	139
Total link	301	532	281	357	385	1,336	137	622
Positive link	88	183	51	117	119	38	6	129
Negative link	213	349	230	240	266	1,298	131	493
Connectance	0.035	0.052	0.042	0.017	0.204	0.153	0.153	0.081
Web asymmetry	–0.481	–0.327	–0.049	–0.237	–0.562	0.027	–0.067	0.376
Links per species	1.42	2.49	1.713	1.194	3.667	7.144	2.283	0.291
No. of compartments	13	8	20	26	3	1	2	1
Specialization asymmetry	0.16	0.079	0.028	0.095	0.107	0.079	–0.026	–0.131
Modularity	0.593	0.46	0.533	0.787	0.276	0.269	0.423	0.527
No. of modules	23	17	28	36	6	5	4	5

### Mining of Keystone Fungal Genus With Biocontrol Potential Against Bacterial Wilt Pathogen

**FIGURE 5:**
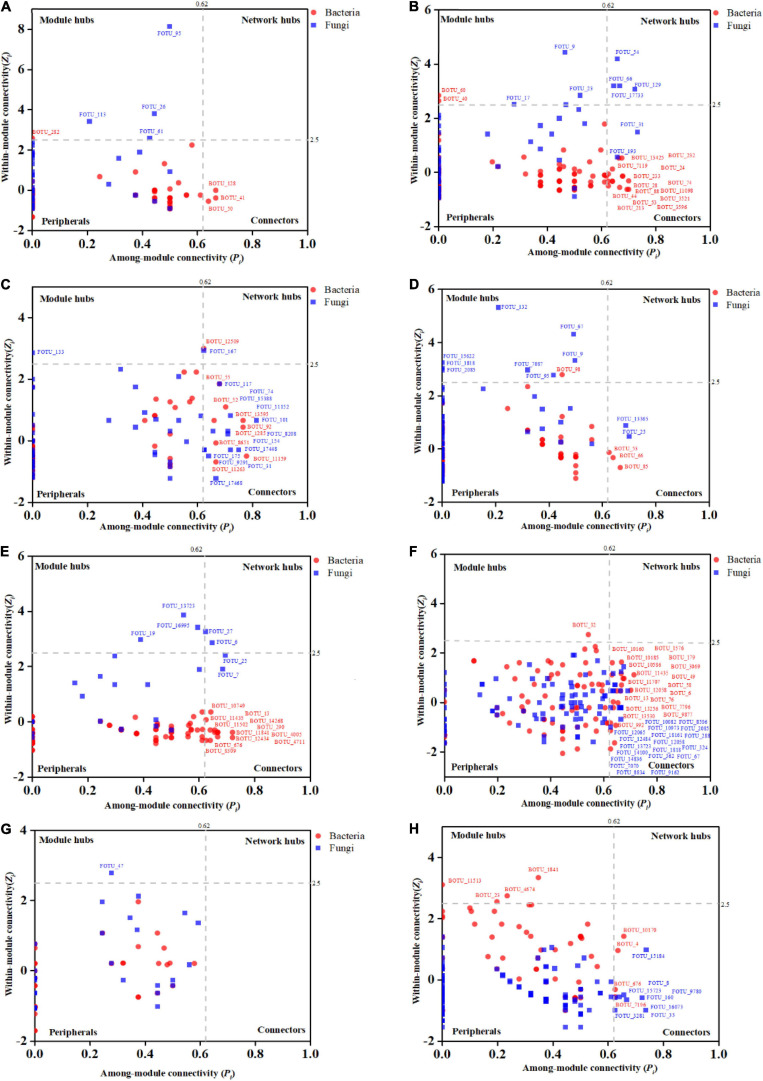
The Zi-Pi plots exhibit the distributions of OTUs based on their topology. The symbols represent OTUs in the bacterial (red dots) and fungal (blue squares) networks. The threshold values of Zi and Pi for categorizing OTUs are 2.5 and 0.62, respectively. **(A)** Healthy bulk soil. **(B)** Infected bulk soil. **(C)** Healthy rhizosphere soil. **(D)** Infected rhizosphere soil. **(E)** Healthy root endophytic compartment. **(F)** Infected root endophytic compartment. **(G)** Healthy stem endophytic compartment; **(H)** Infected stem endophytic compartment.

The Zi-Pi plots were drawn to exhibit the distribution of network nodes OTUs based on the modular topology (Figure 5). The topology of each node (genus) could be measured by its within-module connectivity (*Zi* = 2.5) and among-module connectivity (*Pi* = 0.62). According to the simplified categorization, all nodes in the networks were distributed among four subcategories: Peripherals, Connectors, Module hubs and Network hubs. Nodes that belonged to the latter three subcategories were considered to be keystone microbial microorganisms playing a critical role in the network (Zhou et al., 2011; Jiang et al., 2015) and were marked in the corresponding networks. Interestingly, the bacterial wilt pathogenic *Ralstonia* members were found to be the keystone genus in the IDENs of the infected root and stem endophytic compartments.

**FIGURE 6:**
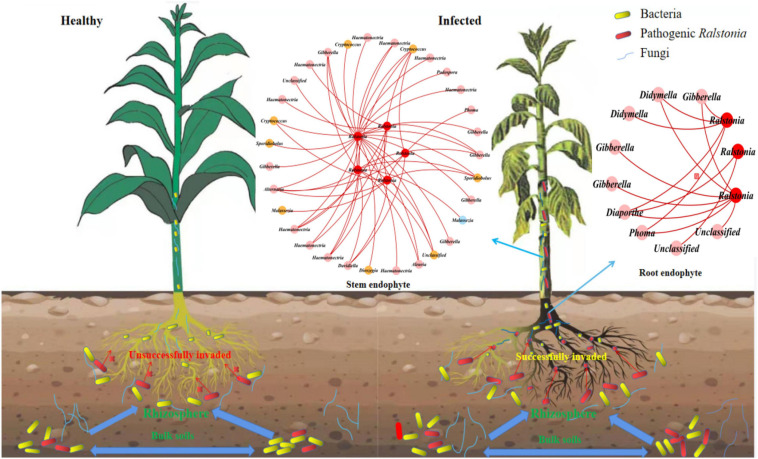
Entangled webs of fungi-bacteria and fungi-pathogen associations in the microbial communities of the surrounding soil, rhizosphere, and root and stem endophytic compartments. The healthy plant **(left)** successfully suppressed invasion of pathogenic *Ralstonia*. The infected **(right)** plant was successfully invaded by the pathogen. The Interdomain ecological networks of the associations between pathogenic bacteria (red) and other fungal species in the root and stem endophytic compartments of the infected samples. Each node is marked at the genus level. Red links indicate negative correlations.

To find the fungal genus closely associated with *Ralstonia* members, we further analyzed the associations between *Ralstonia* members and fungal genus in IDENs for the infected root and stem endophytic compartments. The results showed that the fungal genera *Phoma, Gibberella, Alternaria, Haematonectria, Cryptococcus, Podospora, Spodiobolus, Malassezia, Aleuria, Dioszgia, Davidiealla*, and unclassified genera were negatively correlated with the pathogenic *Ralstonia* members in the root endophytic communities (Figure 6), and the fungal genera *Phoma*, *Gibberella, Diaporthe, Didymella*, and unclassified genera were negatively correlated with *Ralstonia* members in the stem endophytic communities (Figure 6). No positive correlations between fungal genus and the pathogen were found in the root and stem endophytic communities.

## Discussion

The occurrence of plant diseases is closely related to the microbial diversity in the soil and endophytic compartments (Shi et al., 2019; Ulloa-Munoz et al., 2020). Our previous studies have shown that the diversity of endophytic bacterial communities in the roots and stems of plants with bacterial wilt infected was significantly higher than that of healthy samples (Hu Q.L. et al., 2020). In this study, there was no significant difference in the diversity of the root endophytic fungal community between infected and healthy samples. Endophytic fungal community diversity of the infected stem samples was significantly higher than that of the healthy samples. This may be explained by that the bacterial wilt pathogen invasion success within root endophyte could result in the destruction of plant’s defense system. The bacterial wilt pathogen and fungal members interacted closely in the roots, inducing more fungal communities to migrate into the stem, and therefore resulted in an increased diversity of the stem endophytic fungal community compared to the healthy samples (Tan and Zou, 2001; Thebault and Fontaine, 2010; Kefi et al., 2012). Moreover, high diversity and close associations among various microorganism are beneficial to the stability of microbial communities, thereby boosting the microbial community’s resistance to pathogen invasion (McCann, 2000; Wehner et al., 2010; van Elsas et al., 2012; Mallon et al., 2015). The increased diversity of fungal communities in stem endophytes may be a middle-late-stage immune response of the plant to the bacterial wilt pathogen invasion.

The invasion of bacterial wilt pathogen may cause changes in the fungal community composition of the various zones of the tobacco microecosystem. From the perspective of relative abundance, fungal composition displayed significant changes at the genus level between infected and healthy samples in the bulk soil, rhizosphere soil, and root and stem endophytic compartments. In the infected bulk soil and rhizosphere soil, the relative abundances of *Rhodotorula, Ceratobasidium, Cyberlindnera, Podospora, Conocybe, Monoblepharis, Paraconiothyrium*, and *Phoma* were significantly decreased, whereas the relative abundances of *Gibberella*, *Cryptococcus, Mucor, Nectria, Debaryomyces*, and *Haematonectria* were significantly increased, compared to the corresponding healthy samples. Such changes in composition might be because the invasion of bacterial wilt disease made pathogenic *Ralstonia* members the dominant species in soil and thus altered the composition of the soil fungal community. In the infected endophytic samples, the genera that significantly declined were *Cryptococcus, Didymella, Mortierella, Paraphoma, Davidiealla, Phoma*, and *Mucor*. Many of the secondary metabolites produced by these endophytic fungi have been reported to have inhibiting or antibacterial abilities (Melo et al., 2014; Xia et al., 2015; Li et al., 2018). These results indicated that the beneficial endophytes were either actively repelled by the host immune system or defeated by the more dominant migrating microbial community (Lundberg et al., 2012; Velásquez et al., 2017). The relative abundances of *Haematonectria, Gibberella, Ceratobasidium, Nectria, Bionectria*, and *Didymella* were significantly enhanced in the infected endophytic samples, indicating that they may benefit during the pathogen invasion process. It is possible they are opportunists that took advantage of the potential niche opened by pathogen invasion and entered the plant endophytic compartment (Lundberg et al., 2012). The compositional changes of these fungal communities may be caused by changes in root exudates or complex changes in the plant immune system during pathogen invasion (Martinoia and Baetz, 2014; dos Santos et al., 2020), and this promoted the differential recruitment and/or differential rejection of microorganisms to resist the invasion of bacterial wilt pathogen in plant roots and stems (Kwak et al., 2018).

Microbe-microbe associations are essential for the function of microecosystems in soil and endophytic compartments (Barberán et al., 2012). Molecular ecological network (MEN) analysis has been increasingly employed to explore potential microbial associations in various ecosystems (de Menezes et al., 2015). However, there are few reports on microbial associations in the fungal community of plants under invasion by bacterial wilt pathogen. In this study, we applied network analysis to quantify and visualize the associations among microorganisms of the fungal community under the invasion of *R. solanacearum*. The results showed that the fungal networks of infected samples had higher complexity and more links than the healthy samples in the rhizosphere soil and root and stem endophytic compartments. Furthermore, the corresponding topological structures demonstrated significant differences as well. Together this indicated that the invasion of bacterial wilt pathogen changed the composition of the soil fungal community and further strengthened the associations among species in the fungal community. The highly connected and modularized fungal community associations were conducive to regulating the stability of the community (Eisenhauer et al., 2013; Downing et al., 2014; Tardy et al., 2014), thereby controlling the propagation and colonization of the pathogen. Hence, it is necessary to study the associations of microorganisms in soil and endophytic fungal communities for more effective prevention and control of diseases.

Soil is one of the main habitats for bacteria and fungi (Effmert et al., 2012). Endophytic microorganisms, including fungi and bacteria, live in the intercellular or intracellular spaces of plant tissues. The associations between fungi and bacteria are part of the communication network maintaining the balance of this microhabitat (Bamisile et al., 2018). We adopted IDENs to analyze the association network between fungi and bacteria in each zone of the plant-soil microecosystem, and found more complex and tighter fungal-bacterial associations in the infected samples than the corresponding healthy samples for all tested zones. In addition, the IDEN of the infected root endophytic compartment presented the most network links and the highest number of links per microorganism, suggesting a closer bacterial-fungal associations in this network. Interestingly, the Zi-Pi plots demonstrated that the pathogenic *Ralstonia* members were the keystone genus in the root and stem endophytic bacterial-fungal association networks. The reason for these results may be that with the invasion of the bacterial wilt pathogen, more soil fungi and bacteria developed a mutually beneficial relationship and entered the plant root endophytic community together, resulting in more complex associations among microorganisms (Hu Q.L. et al., 2020). It may also be that the competition of nutrient resources or niche space caused more diversified associations between fungi and bacterial microorganisms (Ghoul and Mitri, 2016). A third possibility is that because the microecological balance was broken by the pathogen invasion, leading to more intense antagonistic relationships between fungal, other bacterial members, and the pathogen (García-Bayona and Comstock, 2018; Hu J. et al., 2020). These phenomena were more prominent in infected root and stem endophytic compartments.

To further clarify which fungi interacted closely with the pathogenic *Ralstonia* members in the endophytic roots and stems, we built sub-networks centered on the pathogen, *Ralstonia* members, and included its associations with fungi. The results showed that the root endophytic fungal genera *Phoma, Gibberella, Alternaria, Haematonectria, Cryptococcus, Podospora, Spodiobolus, Malassezia, Aleuria, Dioszgia*, and *Davidiealla*, and the stem endophytic fungal genera *Phoma, Gibberella, Diaporthe*, and *Didymella* were all negatively correlated with *Ralstonia* members. Plant-associated endophytic fungi are rich sources of novel bioactive and structurally diverse secondary metabolites and other natural products, which were generally considered to protect their host plants by blocking or inhibiting the appropriate pathogenic microorganisms (Rustamova et al., 2020). According to previous research, the active compound named as barceloneic acid C isolated and purified from the secondary metabolites of the endophytic fungus *Phoma* sp. JS752, isolated from *Phragmites communis* Trinius, demonstrated an antibacterial activity against pathogenic gram-positive bacteria *Listeria monocytogenes and Staphylococcus pseuditermedius*, and gram-negative bacteria such as *Escherichia coli and Salmonella typhimurium* (Xia et al., 2015). The purified secondary metabolites [(3S)-3,6,7-trihydroxy-α-tetralone, Cercosporamide, β-Sitosterol and trichodermin] of *Phoma* sp. ZJWCF006, which was screened and isolated from the *Arisaema erubescens* endophytes, showed remarkable antibacterial activity against four plant fungal pathogens (*Fusarium oxysporium, Rhizoctonia solani, Colletotrichum gloeosporioides, and Magnaporthe oryzae*) and two plant bacterial pathogens (*Xanthomonas campestris* and *Xanthomonas oryzae*) (Wang et al., 2012). The secondary metabolity compound ergosterol peroxide from the endophytic fungus *Gibberella moniliformis* JS1055, isolated from a halophyte *Vitex rotundifolia* (Kim et al., 2018), exhibited moderate inhibitory activity against bacteria *Staphylococcus aureus* and *Escherichia coli* (Zhu et al., 2017). The endophytic fungus *Alternaria alternata* AE1, isolated from *Azadirachta indica* A. Juss, could produce highly effective bioactive metabolites that showed a strong inhibitory effect on pathogenic bacteria *Listeria monocytogenes* and *Escherichia coli* (Chatterjee et al., 2019). The antibacterial activities of compounds phomosines A and C produced by endophytic fungus *Diaporthe* sp. F2934, isolated from the tropical plant *Aegle marmelos*, showed an antibacterial activity against a variety of gram-negative and gram-positive bacteria, and its inhibitory zone diameter (IZD) against *Staphylococcus aureus* was 20% larger than the standard antibiotic vancomycin (Sousa et al., 2016). It can be seen that these fungi revealed by our study and their secondary metabolites have been reported with antibacterial ability or activity against some bacteria, and they may have potential resistance to bacterial wilt pathogen invasion.

## Conclusion

The bacteria wilt pathogen *Ralstonia* members and the infected root endophytic fungal genera *Phoma, Gibberella, Alternaria, Haematonectria, Cryptococcus, Podospora, Spodiobolus, Malassezia, Aleuria, Dioszgia*, and *Davidiealla*, as well as the infected stem endophytic fungal genera *Phoma, Gibberella, Didymella*, and *Diaporthe*, were negatively correlated, and these fungi may be potential biocontrol resources in dealing with tobacco bacterial wilt disease. At present, there are few reports on the exploration and application of tobacco endophytic fungal resources. This study will provide potential ideas and theoretical support for enriching the study of tobacco endophytic fungal resources and controlling tobacco bacterial wilt disease. Further experiment with species isolation and verification is needed to confirm these findings.

## Data Availability Statement

16S rRNA and ITS gene sequencing data of all samples were submitted to the NCBI SRA database (https://www.ncbi.nlm.nih.gov/) under accession numbers PRJNA540089 and PRJNA735450, respectively.

## Author Contributions

LT, WZ, YX, and SG performed the main experiments and analyzed data. LT, YD, and QH planned and designed the research, wrote the manuscript with substantial input from PL, SW, ZZ, and KF. All authors have read and agreed to the published version of the manuscript.

## Conflict of Interest

The authors declare that the research was conducted in the absence of any commercial or financial relationships that could be construed as a potential conflict of interest.

## Publisher’s Note

All claims expressed in this article are solely those of the authors and do not necessarily represent those of their affiliated organizations, or those of the publisher, the editors and the reviewers. Any product that may be evaluated in this article, or claim that may be made by its manufacturer, is not guaranteed or endorsed by the publisher.

## Footnotes

1
http://mem.rcees.ac.cn:8080

2
http://ieg4.rccc.ou.edu/mena

3
http://mem.rcees.ac.cn:8081
