# Supplementary material for: Fungi-Bacteria Associations in Wilt Diseased Rhizosphere and Endosphere by Interdomain Ecological Network Analysis
Source: Front Microbiol. 2021 Sep 6;12:722626. doi: 10.3389/fmicb.2021.722626 (PMC8450586; doi:10.3389/fmicb.2021.722626)
Supplement: Supplementary file 1 [file Data_Sheet_1.docx]

Supporting information for

**Fungi-bacteria associations in wilt diseased rhizosphere and endosphere by interdomain ecological network analysis**

Lin Tan ^1^, Wei-ai Zeng ^3^, Yansong Xiao ^4^, Pengfei Li ^5^, Songsong Gu ^2,7^, Shaolong Wu ^6^, Zhengguang Zhai ^3^, Kai Feng ^2, 8^, Ye Deng ^2,7,8†^, Qiulong Hu ^1†^

^1^Hunan Agricultural University, Changsha, Hunan, China

^2^CAS Key Laboratory for Environmental Biotechnology, Research Center for Eco-Environmental Sciences, Chinese Academy of Sciences, Beijing, China

^3^Changsha Tobacco Company of Hunan Province, Changsha, Hunan, China

^4^Chenzhou Tobacco Company of Hunan Province, Chenzhou, Hunan, China

^5^Wenshan Tobacco Company of Yunnan Province, Wenshan, Yunnan, China

^6^Tobacco Company of Hunan Province, Changsha, Hunan, China

^7^Institute for Marine Science and Technology, Shandong University, Qingdao, China

^8^College of Resources and Environment, University of Chinese Academy of Sciences, Beijing, China

^††^**Co-author for correspondence:** Ye Deng, Email: yedeng@rcees.ac.cn. Qiulong Hu, Email: huqiulongnet@126.com

Table S1 Description of sampling 5 tobacco field sites

| Sample Location  (Guiyang Country) | Longitude | Latitude | Altitude | Soil physics | I[nfection Grade](C:/Program%20Files%20(x86)/Youdao/Dict/8.9.6.0/resultui/html/index.html#/javascript:;) | No. of Samples | | | |
| --- | --- | --- | --- | --- | --- | --- | --- | --- | --- |
|  |  |  |  |  |  | Bulk soil | rhizosphere | Root | Stem |
| Haotang Town | 25°47′19.01″ | 112°37′14.59″ | 216 | Clay | 0 | 2 | 2 | 2 | 2 |
|  |  |  |  |  | 7 | 2 | 2 | 2 | 2 |
| Leiping Town | 25°45′34.28″ | 112°41′51.31″ | 225 | Loam | 0 | 2 | 2 | 2 | 2 |
|  |  |  |  |  | 5 | 2 | 2 | 2 | 2 |
| Heping Town | 25°56′10.81″ | 112°33′41.85″ | 245 | Clay | 0 | 2 | 2 | 2 | 2 |
|  |  |  |  |  | 9 | 2 | 2 | 2 | 2 |
| Tangshi Town | 25°47′43.97″ | 112°22 41.14″ | 220 | Loam | 0 | 2 | 2 | 2 | 2 |
|  |  |  |  |  | 7 | 2 | 2 | 2 | 2 |
| Renyi Town | 25°51′16.46″ | 112°24′12.76″ | 189 | Clay | 0 | 2 | 2 | 2 | 2 |
|  |  |  |  |  | 7 | 2 | 2 | 2 | 2 |

Table S2 Relative abundance of dominant phylum and genera in soil and plant endophytic commuties from healthy and infected samples. HBS: bulk soils samples of healthy tobacco, IBS: bulk soil samples of wilt-infected tobacco, HRS: rhizosphere samples of healthy tobacco, IRS: rhizosphere samples of wilt-infected tobacco, HR: root samples of healthy tobacco, IR: root samples of wilt-infected tobacco, HS: stem samples of healthy tobacco, IS: stem samples of wilt-infected tobacco.

| **Domain phyla** | Relative abundance (%) | | | | | | | |
| --- | --- | --- | --- | --- | --- | --- | --- | --- |
|  | HBS | IBS | HRS | IRS | HR | IR | HS | IS |
| *Ascomycota* | 64.01ab | 50.42b | 60.36ab | 66.59ab | 81.12a | 71.5ab | 74.32a | 73.99a |
| *Basidiomycota* | 18.13a | 16.61a | 18.61a | 16.06a | 15.04a | 28.00a | 25.02a | 25.66a |
| *Chytridiomycota* | 3.13a | 0.27c | 2.70ab | 1.51bc | 0.09c | 0.01c | 0.00c | 0.01c |
| *Glomeromycota* | 0.09ab | 0.03b | 0.06ab | 0.02ab | 0.17a | 0.05ab | 0.02ab | 0.01b |
| *Unclassified* | 0.95ab | 0.61b | 1.09ab | 1.13ab | 2.69a | 0.2b | 0.09b | 0.06b |
| *Zygomycota* | 13.69c | 32.06a | 17.17b | 14.70b | 0.90d | 0.24d | 0.55d | 0.27d |
| **Domain genera** |  |  |  |  |  |  |  |  |
| Unclassified | 11.71bc | 14.30bc | 15.19bc | 12.12bc | 31.38a | 24.33ab | 4.54c | 2.90c |
| *Plectosphaerella* | 6.31bc | 2.25c | 4.75bc | 4.99bc | 15.83ab | 1.87c | 14.97ab | 21.15a |
| *Mortierella* | 12.20bc | 23.70a | 17.79ab | 11.43bc | 1.49cd | 0.10d | 0.43d | 0.19d |
| *Paraphoma* | 1.45c | 2.76c | 2.37c | 1.67c | 1.72c | 0.34c | 26.97a | 14.01b |
| *Gibberella* | 2.15c | 5.20ab | 5.54ab | 6.95ab | 4.03ab | 17.21a | 2.64c | 13.45ab |
| *Rhodotorula* | 2.03bc | 1.10bc | 2.17bc | 0.96c | 4.02bc | 0.05c | 15.49ab | 21.59a |
| *Aleuria* | 16.51a | 9.16ab | 7.89ab | 11.48ab | 0.09b | 0.03b | 0.02b | 0.03b |
| *Alternaria* | 0.53b | 1.48b | 0.89b | 0.51b | 4.50ab | 0.15b | 2.86ab | 13.11a |
| *Cryptococcus* | 2.94ab | 8.75a | 3.28ab | 4.57ab | 1.80ab | 0.22b | 5.53ab | 1.82ab |
| *Mucor* | 1.05ab | 7.09a | 1.78ab | 7.05a | 0.79b | 0.10b | 0.79b | 0.07b |
| *Ceratobasidium* | 0.70c | 0.40c | 0.60c | 0.06c | 5.33b | 16.33a | 0.09c | 1.37bc |
| *Nectria* | 0.35b | 0.56b | 0.52b | 1.86ab | 1.96ab | 8.72a | 0.01 | 2.47ab |
| *Davidiella* | 0.38c | 0.37c | 0.46c | 0.24c | 3.32ab | 0.29c | 5.37a | 1.69bc |
| *Cyberlindnera* | 8.38a | 0.72c | 7.40a | 4.33b | 0.09c | 1.88bc | 0.10c | 0.02c |
| *Debaryomyces* | 0.66b | 3.53ab | 0.41b | 6.10a | 0.07b | 0.56 | 1.08ab | 0.19b |
| *Wickerhamomyces* | 0.00b | 0.00b | 0.00b | 0.00a | 0.01b | 0.00b | 9.55a | 0.02b |
| *Haematonectria* | 0.09b | 0.40b | 0.34b | 0.73b | 1.70ab | 7.96a | 0.02b | 0.72b |
| *Podospora* | 2.97a | 1.53ab | 3.21a | 1.83ab | 0.49b | 0.06b | 0.03b | 0.06b |
| *Guehomyces* | 2.50a | 1.35ab | 1.48ab | 1.38ab | 0.10b | 0.01b | 0.10b | 0.08b |
| *Bionectria* | 0.15b | 0.08b | 0.12b | 0.68b | 0.10b | 8.30a | 0.01b | 0.61b |
| *Conocybe* | 1.68ab | 0.28b | 3.18a | 1.72ab | 0.01b | 0.04b | 0.01b | 0.00b |
| *Thanatephorus* | 0.10b | 0.35b | 0.03b | 0.01b | 0.08b | 7.64a | 0.00b | 0.09b |
| *Monoblepharis* | 1.77a | 0.58ab | 1.75a | 0.85ab | 0.04b | 0.01b | 0.09b | 0.00b |
| *Fusarium* | 1.49ab | 0.67b | 0.43b | 2.46a | 0.23b | 0.02b | 0.00b | 0.06b |
| *Didymella* | 0.46b | 0.83b | 0.78b | 0.64b | 0.13b | 0.01b | 1.69a | 0.46b |
| *Paraconiothyrium* | 1.35a | 0.43b | 1.56a | 0.96ab | 1.54a | 0.05b | 0.02b | 0.06b |
| *Phoma* | 0.67b | 0.17b | 0.52b | 0.19b | 1.67ab | 0.06b | 2.49a | 0.21b |
| *Neurospora* | 0.53b | 0.21b | 0.39b | 0.37b | 4.50a | 0.00b | 0.01b | 0.01b |
| *Entoloma* | 3.3a | 0.02b | 0.57b | 1.65ab | 0.62b | 0.01b | 0.00b | 0.00b |
| *Talaromyces* | 0.75ab | 0.94a | 0.64ab | 0.51ab | 0.58ab | 0.04b | 0.01b | 0.03b |
| *Others* | 14.83a | 10.81abc | 13.94a | 11.70ab | 11.80ab | 3.59c | 4.98bc | 3.54c |

Different letters in the table represent significant difference (*p* < 0.05).
